# Dataset on patients with Recurrent Borderline Ovarian Tumors and Table with Review of Literature on Fertility and Oncologic Outcomes of patients with Borderline Ovarian Tumors

**DOI:** 10.1016/j.dib.2020.105653

**Published:** 2020-04-30

**Authors:** Helmut Plett, Enzo Ricciardi, Philipp Harter, Beyhan Ataseven, Florian Heitz, Sonia Prader, Stephanie Schneider, Sebastian Heikaus, Annette Fisseler-Eckhoff, Friedrich Kommoss, Sigurd F. Lax, Annette Staebler, Alexander Traut, Andreas du Bois

**Affiliations:** a Department of Gynecology and Gynecologic Oncology, Ev. Kliniken Essen-Mitte, Essen, Germany; b Department of Gynecology, Charité University Hospital, Berlin, Germany; c Center of Pathology Essen-Mitte, Essen, Germany; d Institute of Pathology, Helios Dr. Horst-Schmidt-Kliniken, Wiesbaden, Germany; e Institute of Pathology, Medizin Campus Bodensee, Friedrichshafen, Germany; f Department of Pathology, LKH Graz II, Graz and Johannes Kepler University, Linz, Austria; g Institute of Pathology and Neuropathology, University of Tuebingen, Germany; h Department of Obstetrics and Gynecology, University Hospital, LMU Munich, Germany

**Keywords:** Borderline tumors of the ovary, Fertility sparing surgery, Radical surgery, Recurrence, Conservative treatment, Invasive Recurrence, Low-grade ovarian cancer, Review of Literature

## Abstract

The data presented here is related to the research article entitled “FERTILITY-SPARING SURGERY AND REPRODUCTIVE-OUTCOMES IN PATIENTS WITH BORDERLINE OVARIAN TUMORS” by Plett et al. in Journal of Gynecologic Oncology [1] and is analysed and discussed in detail. 18 Patients with Recurrent Borderline Ovarian Tumors (BOT) were identified and listed in Table 1. All patients underwent treatment for primary BOT either per radical surgery (RS) or fertility sparing surgery (FSS) by the same team in Horst Schmidt Klinik (HSK) in Wiesbaden and the Department of Gynecology and Gynecologic Oncology at Kliniken Essen-Mitte between January 2000 and December 2018 and were followed up closely. Details on patients` and surgical characteristics are given as well as management of character of recurrent disease. In Table 2 important publications from the last 20 years are listed in order to visualize better the oncologic outcomes (invasive and non-invasive relapses) and calculated risks of recurrence with the purpose to understand better the important findings of the related article cited above.

Specifications Table

**Table:** 

Subject	Obstetrics, Gynecology and Women's Health
Specific subject area	Borderline tumors of the ovary
Type of data	Table
How data were acquired	1. Review of literature and choice of most important articles on Radical – and Fertility Sparing Surgery with detailed information on oncologic and fertility outcome [2], [3], [4], [5], [6], [7], [8], [9], [10], [11], [12].
	2. Identification of patients with Recurrent BOT by close follow up and gathering information on primary and recurrent disease from their clinical files. Instruments: Excell table
Data format	Analyzed
Parameters for data collection	352 Patients with primary diagnosis of BOT treated and documented prospectively in our hospital database.
Description of data collection	All patients were treated by the same team in two locations in Germany: Horst Schmidt Klinik (HSK) in Wiesbaden between January 2000 and December 2010 and the Department of Gynecology and Gynecologic Oncology at Kliniken Essen-Mitte between January 2011 and December 2018
Data source location	City/Region: Essen, Nord-Rhein-Westfalen Country: Germany
Data accessibility	With the article
Related research article	Fertility-sparing surgery and reproductive-outcomes in patients with borderline ovarian tumors. By: Helmut Plett MD^a,b^, Philipp Harter MD, PhD^a^, Beyhan Ataseven MD, PhD^a,h^, Florian Heitz MD^a,b^, Sonia Prader MD^a^, Stephanie Schneider MD^a^, Sebastian Heikaus MD, PhD^c^, Annette Fisseler-Eckhoff^d^, Friedrich Kommoss^e^, Sigurd F. Lax^f^, Annette Staebler^g^, Alexander Traut^a^, Andreas du Bois MD, PhD^a^ DOI:10.1016/j.ygyno.2020.02.007 Gynecologic Oncology

## Value of the data

• These data may be useful for a both better interpretation of data presented in the article related to this publication and to serve for future studies to be compared or included to.
• Researchers in the field of gynecologic oncology and specifically in the fertility preservation
• These data may be useful to strengthen the evidences for fertility preservation in BOT and possibly constitute the basis for the design of future studies.
• We also include a review of recent literature data who might be really helpful in having a thorough view of the state of the art of research in this field.

## 1 Data Description

Supplementary Table 1: Detailed patients` demographics of 5 patients after RS (pat. 1-5) and 13 patients after FSS (pat. 6-18) with recurrent BOT (n=13) or malignant transformation (n=4)

Supplementary Table 2: Recent published data on oncologic outcome after FSS and RS and fertility outcome after FSS. (2-12)

## 2 Experimental Design, Materials, and Methods

From January 2000 to December 2018, 352 Patients with primary diagnosis of BOT were treated and documented prospectively in our hospital database. All patients were subsequently treated by the same team in two locations in Germany: Horst Schmidt Klinik (HSK) in Wiesbaden between January 2000 and December 2010 and the Department of Gynecology and Gynecologic Oncology at Kliniken Essen-Mitte between January 2011 and December 2018. Histopathological examination was performed by local pathologists and additional external histopathological review was carried out in all cases. Cases of BOT with invasive implants before 2014 (n=7) and therefore retrospectively considered as LGSOC, were not excluded in order to make our data comparable to other publications. FSS was performed after individual discussion. Complete surgical staging was defined according to FIGO but without lymphadenectomy and with a waiver for preservation of uterus and one ovary in case of FSS. We divided patients in 2 groups, the FSS-group and the RS-group. The records of patients are updated annually. Patients were followed up by a telephone interview directly or vicariously by their treating general gynecologist and data on recurrent disease as well as data on the reproductive outcome were collected. Pregnancy was defined on the base of ultrasound findings by the treating gynecologist. This study was exempt from an ethic approval process because of his retrospective nature. Patients gave informed consent for documentation of clinical data in the associated clinical tumor registries and for clinical research and have voluntarily agreed on a telephone interview for follow up. Patients from this series were included in prior publications.

### 2.1 Statistical analysis

All statistical analyses were performed using SPSS version 23.0 (IBM Corporation, New York, USA). The Kaplan-Meier method was used for univariate analysis of disease-free survival (DFS). Since only 1 patient had a fatal event we did not include any OS analysis in our manuscript. Different survival curves were compared using the log-rank test. DFS was calculated in months from initial surgery to the date of recurrence. The Cox proportional hazard model (Wald stepwise backward regression) was utilized to evaluate all parameters that were significant in univariate analyses. The multivariate adjusted odds ratios (OR) and 95% confidence intervals (CI) are expressed. All analyses were thought to be hypothesis generating and a p-value of <0.05 was considered being significant.

## Appendix Supplementary materials

Image, application 1

## References

[1] Plett H., et al. under submission (Gynecologic Oncology) “FERTILITY-SPARING SURGERY AND REPRODUCTIVE-OUTCOMES IN PATIENTS WITH BORDERLINE OVARIAN TUMORS” Reference to a journal publication with an article number: (citation numbers as cited in the original article by Plett et al.) 10.1016/j.ygyno.2020.02.007 32115229

[2] Sozen H., Vatansever D., Topuz S., Iyibozkurt C., Kandemir H., Yalçin I., Onder S., Yavuz E., Salihoglu Y. (2018). Clinicopathological analysis of borderline ovarian tumours and risk factors related to recurrence: experience of single institution. J Obstet Gynaecol.

[3] du Bois A., Ewald-Riegler N., de Gregorio N., Reuss A., Mahner S., Fotopoulou C., Kommoss F., Schmalfeldt B., Hilpert F., Fehm T., Burges A., Meier W., Hillemanns P., Hanker L., Hasenburg A., Strauss H.-G., Hellriegel M., Wimberger P., Keyver-Paik M.-D., Baumann K., Canzler U., Wollschlaeger K., Forner D., Pfisterer J., Schröder W., Münstedt K., Richter B., Kommoss S., Hauptmann S., Arbeitsgmeinschaft Gynäkologische Onkologie (AGO) Study Group (2013). Borderline tumours of the ovary: A cohort study of the Arbeitsgmeinschaft Gynäkologische Onkologie (AGO) Study Group. Eur J Cancer.

[4] Lou T., Yuan F., Feng Y., Wang S., Bai H., Zhang Z. (2017). The safety of fertility and ipsilateral ovary procedures for borderline ovarian tumors. Oncotarget.

[5] Delle Marchette M., Ceppi L., Andreano A., Bonazzi C.M., Buda A., Grassi T., Giuliani D., Sina F., Lamanna M., Bianchi T., Lissoni A.A., Landoni F., Valsecchi M.G., Fruscio R. (2019). Oncologic and fertility impact of surgical approach for borderline ovarian tumours treated with fertility sparing surgery. Eur J Cancer.

[6] Uzan C., Muller E., Kane A., Rey A., Gouy S., Bendiffallah S., Duvillard P., Fauvet R., Darai E., Morice P. (2014). Prognostic factors for recurrence after conservative treatment in a series of 119 patients with stage I serous borderline tumors of the ovary. Ann Oncol.

[7] Romagnolo C., Gadducci A., Sartori E., Zola P., Maggino T. (2006). Management of borderline ovarian tumors: results of an Italian multicenter study. Gynecol Oncol.

[8] Vancraeynest E., Moerman P., Leunen K., Amant F., Neven P., Vergote I. (2016). Fertility Preservation Is Safe for Serous Borderline Ovarian Tumors. Int J Gynecol Cancer.

[9] Song T., Lee Y.-Y., Choi C.H., Kim T.-J., Lee J.-W., Bae D.-S., Kim B.-G. (2014). Risk factors for progression to invasive carcinoma in patients with borderline ovarian tumors. Int J Gynecol Cancer.

[10] May J., Skorupskaite K., Congiu M., Ghaoui N., Walker G.A., Fegan S., Martin C.W., O'Donnell R.L. (2018). Borderline Ovarian Tumors: Fifteen Years’ Experience at a Scottish Tertiary Cancer Center. Int J Gynecol Cancer.

[11] Zanetta G., Rota S., Lissoni A., Meni A., Brancatelli G., Buda A. (2001). Ultrasound, physical examination, and CA 125 measurement for the detection of recurrence after conservative surgery for early borderline ovarian tumors. Gynecol Oncol.

[12] Park J.-Y., Kim D.-Y., Kim J.-H., Kim Y.-M., Kim Y.-T., Nam J.-H. (2009). Surgical management of borderline ovarian tumors: The role of fertility-sparing surgery. Gynecol Oncol.

