# Supplementary material for: Dataset on patients with Recurrent Borderline Ovarian Tumors and Table with Review of Literature on Fertility and Oncologic Outcomes of patients with Borderline Ovarian Tumors
Source: Data Brief. 2020 Apr 30;30:105653. doi: 10.1016/j.dib.2020.105653 (PMC7206201; doi:10.1016/j.dib.2020.105653)
Supplement: Supplementary file 1 [file mmc1.zip › Supplementary Table 2_BOT.docx]

Table 2: Recent published data on oncologic outcome after FSS and RS and fertility outcome after FSS.

|  | KEM 2019 | Delle Marchette et al.  2019 [17] | Sozen 2018  [7] | May et al. 2018  [24] | Lou et al.  2017 [13] | Vancraeynest et al.  2016 [20] | Uzan et al. 2013 [18] | ROBOT  du Bois et. al. 2013 [9] | Song et al.  2011 [21] | Park et. al.  2009 [31] | Romagnolo et. Al.  2006 [19] | Zanetta et al.  2001 [29] |
| --- | --- | --- | --- | --- | --- | --- | --- | --- | --- | --- | --- | --- |
| N total | 352 | 535 | 103 | 275 | 281 | 132 (SBOT) | 119 SBOT - FIGO I | 950 | 298 | 360 | 113 | 339 |
| FSS | 95 | 535 | 40 | 58 | 138 | 42 | 119 | 166 | 155 | 184 | 53 | 189 |
| Relapses after FSS | 13 | 228 | 13 | 5 | 17 | 8 | 38 | 39 | 12 | 9 | 9 | 35 |
| Overall-RR (med. follow-up period) | 13.5% (63 months) | 34%  (162 months) | 32.5% (50 months) | 8.6% (50 months) | 12.3% (43 months) | 19% (6 years) | 32%  (43 months) | 23.5% | 7.7% (54.4months) | 5% (70 months) | 17% (44 months) | 18% (70 months) |
| Invasive Relapses | 0 | 8 (1.5%) | n.a. | 2 (3.4%)  1 DOD | n.a.  (2 Pat. – DOD) | 1  (2.4%) | 2 (1.68%) | 2  (1.2%) | 1 | 4 | n.a. | n.a. |
| Incidence of 1^st^ pregnancy after FSS | 82.9% | 84.6% | n.a. | n.a. | n.a. | n.a. | n.a. | n.a. | 88% | 87% | 67% | n.a. |
| RS | 257 | n.a. | 63 | 217 | 143 | 90 | n.a. | 784 | 143 | 176 | 60 | 150 |
| Relapses after RS | 5 |  | 3 | 7 | 3 | 6 |  | 36 | 7 | 9 | 4 | 7 |
| Overall-RR (median follow-up) | 1.9% (63 months) |  | 4.8% (50 months) | 3.2% (50 month) | 3.2% (50 months) | 6.7% (6 years) |  | 4.6% | 4.9% (54.4 follow-up) | 5.1% (70 months) | 7% (44 months) | 5% (70 months) |
| Invasive Relapses | 4 (1.55%)  1 DOD |  | n.a. | 5 (2.3%)  4 DOD | n.a.  (2 Pat. DOD) | 4 (4.4%)  1 DOD |  | 20 (2.6%) | 3 (2.1%) | 4 (2.3%)  4 DOD | n.a. | n.a. |

SBOT – serous borderline tumor

FSS – fertility sparing surgery

RR – risk of recurrence

RS – radical surgery

DOD – died of disease

n.a. – not available
