# Supplementary material for: Dataset on patients with Recurrent Borderline Ovarian Tumors and Table with Review of Literature on Fertility and Oncologic Outcomes of patients with Borderline Ovarian Tumors
Source: Data Brief. 2020 Apr 30;30:105653. doi: 10.1016/j.dib.2020.105653 (PMC7206201; doi:10.1016/j.dib.2020.105653)
Supplement: Supplementary file 1 [file mmc1.zip › Supplementary Table_1_14.11.2019.docx]

Table 1: Detailed patients` demographics of 5 patients after RS (pat. 1-5) and 13 patients after FSS (pat. 6-18) with recurrent BOT (n=13) or malignant transformation (n=4)

| Pat.  Nr. | Age  (yr) | CA 125  (U/ml) | Radical Surgery Group  Primary Surgery (PS)  Completion Surgery (CS) | Histology | Micro-inva-sion | FIGO | LNE pelv  paa | DFS (m) | CA 125 at rec. | Location of recurrence | Surgical management of recurrence | Recurrence; malignant Transformation; FIGO-Stage  Further recurrences | Pat. Status | Follow-up (m) |
| --- | --- | --- | --- | --- | --- | --- | --- | --- | --- | --- | --- | --- | --- | --- |
| 1 | 29 | n.a. | PS - LSC: (09/07) exploratory biopsies  PS - LT: (09/07) peritoneal biopsies  CS - LT: (11/07) HE, BSO, AE, deperit. of pelvis and right diaphragma, RD=0 | papillary-serous | No | IIIB | X | 29 | 460 | bursa, stomach, small oment. pelvis | LT: (02/10) resection of the omentum minus, deperitonealisation of the left diagphragma, OE, pelvic tumor resection, RD=0 | SBOT | NED | 119 |
| 2 | 44 | n.a. | LT: (07/07) HE, BSO, OE, AE, pelvic and paracholic, deperitonealisation  RD = 0 | SBOT (with invasive implants) | No | IIIB | X | 47 | 93 | vaginal trunc | LT: (10/11) pelvic and diaphragmatic deperit., left hemicolectomy, resection of the vaginal trunc, resection of the liver capsula, RD=0 | **LGSOC**, FIGO IIIB, 1st-line Carboplatin/Paclitaxel, Bevaci-zumab ongoing since 2011,  no further recurrence | NED | 120 |
| 3 | 68 | n.a. | LT: (12/11) BSO, previous HE, deperit., OE, resection of the sigmoid (descendorectostomy), ileocecal resection (ileoascendostomy), RD=0  (2^nd^ malignancy: simult. neuroendocrin tumor of the ileum, G1, pT3, pN0, M0 ; Carcinoid) | SBOT | Yes | IIIB | 0/9 | 15 | 87 | Right pelvic wall  Pelvic  lymph. nodes | LT: (04/13) Resection of tumor in the pelvic wall, pelvic and inguinal LNE (bilat.), pelvic deperitonealisation, RD = 0 | **LGSOC** FIGO IIIC  pN1 (3/7)  1st line 6 cycles Carboplatin/Paclitaxel + Bevacizumab | NED | 69 |
| 4 | 50 | n.a. | PS - LSC (08/02) left USO, right salpingectomy  CS - LT: (03/03) HE, right USO, pelvic / paraaortic LNE,  RD=0 | Psammo-matous | Yes | IIIA1 | 2/  38 | 38 | 36 | Appendix and left pelvic wall | LR: (11/05) AE, Tumor resection left and right pelvic wall, RD = 0 | **LGSOC** FIGO IIIC (psamm.)  2^nd^ 03/2010 LT: left hemicolectomy, peritoneal tumor resection, RD = 0 | NED | 162 |
| 5 | 64 | n.a. | LT: (07/01) HE, BSO, pelv. lymph. nodes, OE, perit. biopsies, cholecystectomy, RD=0 | SBOT | No | IIIB | 1/  X | 89 | n.a. | Sigmoid, abd. lymph. nodes, abdominal wall, | LT: (12/08) resection of sigmoid, TE abdominal wall (RD=1 / R1) | Serous carcinoma;  (**LGSOC** FIGO IVB)  1^st^ line 3 cycles Carboplatin  03/09 Staging: PD  2^nd^ line 3 cycles of caelyx mono  08/10 Staging PD | DOD  05/11 | 118 |
| Pat.  Nr. | Age  (yr) | CA 125  (U/ml) | Fertility Sparing Surgery Group  Primary Surgery (PS)  Completion Surgery (CS) | Histology | Micro-inva-sion | FIGO | LNE pelv  paa | DFS (m) | CA 125 at rec. | Location of recurrence | Surgical management of recurrence | Recurrence; malignant Transformation; FIGO-Stage  Further recurrences | Pat. Status | Follow-up (m) |
| 6 | 25 | 61 | PS - LSC: (04/14) Left USO, Peritoneal biopsy;  CS - LT: (09/14) pelvic peritonectomy, contralat. ovarian biopsy, AE, pelvic node excision, RD=0 | SBOT | No | IIIB | 0_1 pelv | 18 | n.a. | contralateral (right) ovary | LSC: (03/16) Cystectomy right ovary, salpingectomy (FSS) | serous-mucinous BOT;  2^nd^ recurrence (10/17) TLH, right USO, RD = 0 | NED | 51 |
| 7 | 19 | 68 | Diagnostic LSC: (02/14) biopsy  CS - LT: (02/14) OE, splenectomy, AE, pelvic and bilat. diaphragmatic deperit., left USO, resection of sigmoid, paraaortal node sampling  RD=0 | SBOT | No | IIIB | 0_2  Paraaortal | 49 | n.a. | contralateral (right) ovary, serosa of the uterus, pelvic serosa | LSC: (01/18) right ovarian cystectomy  LT: (05/18) right USO, pelvic peritonectomy, RD=0 | SBOT  rpT2a  No further recurrence | NED | 53 |
| 8 | 33 | n.a. | LT (01/10) left USO, OE, AE, peritoenal biopsy, Tumorectomy right ovary, (FSS) TR=0 | SBOT | No | IIIB | X | 32 | n.a. | contralateral (right) ovary | LT: (09/12) HE, right USO, peritoneal biopsy, RD=0 | SBOT rpT1a,  No further recurrence | NED | 79 |
| 9 | 28 | n.a. | PS - Diagnostic LSC: (03/06) peritoneal biopsy  CS - LT: (03/06) right USO, left ovarian biopsy, OE, peritoneal biopsy, (FSS) RD=0 | SBOT | No | IC2 | X | 13 | n.a. | contralateral (left) ovary | Cesarian section: (04/07), biopsy left ovary  LT: (10/07) HE, left USO, pelvic and paracolic peritonectomy, RD=0 | serous-papillary BOT,  No further recurrence | NED | 153 |
| 10 | 35 | n.a. | PS - LSC: (07/09) right ovarian cystectomy  CS - LT: (12/09) right USO, left ovarian biopsy, OE, peritoneal biopsy, AE, (FSS) RD = 0 | SBOT | No | IC3 | X | 87 | 25 | contralateral (left) ovary | (07/11 natural birth)  LSC: (10/16) HE, left USO, peritoneal biopsy, Peritoneal PE, RD = 0 | SBOT, rpT1a  No further recurrence | NED | 112 |
| 11 | 25 | 105 | PS - LSC: (05/16) ovarian cystectomy bilat., pelvic peritoneal biopsy  CS - LT: (06/16) deperit. of the pelvis and right diaphragma, OE, AE, superficial tumor resection of ovaries bilat., (FSS) RD=0 | SBOT | No | IIIB | X | 6 | n.a. | Left and right ovary, Douglas | (Kryoconservation of oocytes)  LT: (11/16) BSO, biopsy of the sigma, douglas, pelvic peritoenum, serosa of the uterus, RD=0 | SBOT  rpT2b  No further recurrence | NED | 30 |
| 12 | 24 | n.a. | PS- LSC: (10/04) right ovarian cystectomy, left ovarian biopsy  CS - LSC: (11/04) left USO, peritoneal biopsy, omental biopsy, sampling of pelvic nodes, AE  CS-LT: (02/05)  OE, pelvic depariton., right diaphragma, TE left pelvic wall, (FSS), RD=0 | serous (diploid)  BOT | No | IIIB | 0/7 | 141 | 18 | contralateral right ovary | 07/16: LT, right USO, RD=0 | SBOT  No further recurrence | NED | 169 |
| 13 | 21 | n.a. | LT: (08/02) right USO, cystectomy left ovary, OE, paraaortal bulky node exzision, (FSS) RD=0 | SBOT | No | IIIA2 | 0/3 | 120 | n.a. | contralateral (left) ovary | LT: (08/12) HE, left USO, RD=0 | SBOT  No further recurrence | NED | 195 |
| 14 | 30 | n.a. | Diagnostic LSC: (11/01) Biopsy  LT: (12/01) right USO, left ovarian biopsy, deperit. Douglas, OE, (FSS) RD=0 | SBOT | No | IIB | 0 | 13 | <35 | Bladder serosa, left ovary | LT: 01/03 left SO, pelvic deperitonealisation, RD = 0  No further recurrence | papillary-serous BOT,  No further recurrence | NED | 203 |
| 15 | 29 | 20 | LSC: (02/04) left USO,  LT: (03/04) right ovarian biopsy, OE  peritoneal biopsy Douglas, bladder diaphragma bilat., RD=0 (FSS) | papillary-serous  BOT | No | IIIB | X | 8 | 20 | right ovary | LT: (09/04) right SO, peritoneal biopsy of douglas and bladder, RD = 0 | papillary-serous BOT,  No further recurrence | NED | 178 |
| 16 | 20 | 52 | LSC: (04/11) ovarian bilat. cystectomy  Staging-LSC: 06/11 infracolic OE, deperit. of the right diaphragma, peritoneal biopsy, (FSS) RD=0 | papillary-serous  BOT | No | IIIA2 | X | 70 | 63 | Right ovary Douglas, right paracolic space | LT: (02/17) right USO, deperitonealisation pelvic walls, exzision bulky node left iliaca; RD = 0 (FSS) | SBOT  2nd recurrence; LT 06/18 cystectomy left ovary,  RD=0, FSS | NED | 89 |
| 17 | 21 | 460 | LT: (03/12) right USO,  omentum biopsy, peritoneum of the bladder and diaphragma bilat.,  Staging-LT: 04/12 OE, deperitonealisation diaphragma and pelvis (FSS), RD=0 | SBOT | Yes | IIB | X | 37 | 38 | Peritoneum of the bladder and left pelvic wall | LT: (05/15) Cystectomy left ovary, deperitonealisation of the bladder and left pelvic wall, RD=0 (FSS) | BOT (adenofibromatous type) with microinvasion;  No further recurrence | NED | 79 |
| 18 | 37 | n.a. | LSC: (04/17): left USO, cystectomy right ovary  LSC Staging: (08/17) OE, AE, right salpingectomy, deperitonealisation pelvic walls, RD=0 | MBOT | No | IC1 | X | 15 | n.a. | Right ovary | LT: (11/18), right USO, RD=0 | MBOT | NED | 24 |

DFS: disease free survival,

LSC = laparoscopy; LT = laparotomy,

PS = primary surgery; CS = completing surgery;

AE = appendectomy; OE = omentectomy; TE tumorectomy;

HE = hysterectomy; TLH = total laparoscopic hysterectomy,

USO = unilateral salpingo-oophorectomy; BSO = bilateral salpingo-oophorectomy;

RD = residual disease;

FSS = fertility sparing surgery;

NED = no evidence of disease; AWD = alive with disease; DOD = died of disease;

LGSOC = low grade serous ovarian cancer; PD = progressive disease;

SBOT = serous BOT; MBOT = muzinous BOT

n.a. = not available
